# Allograft Screws as Fixation of the Scarf Osteotomy

**DOI:** 10.3390/jcm13185628

**Published:** 2024-09-23

**Authors:** Kevin Döring, Sebastian Apprich, Markus Hanna, Reinhard Windhager, Stephan Puchner

**Affiliations:** Department of Orthopedics and Trauma Surgery, Medical University of Vienna, 1090 Vienna, Austria

**Keywords:** scarf osteotomy, allograft bone screw, headless compression screw

## Abstract

**Background:** In comparison to titanium screws, novel cortical bone allograft screws may come with advantages in osseointegration and with avoidance of potential material removal surgery after scarf osteotomy. **Methods:** A scarf osteotomy with allograft bone screws as fixation was performed in 21 patients (30 feet). Clinical and radiological parameters were prospectively collected until one year after surgery. A retrospective control group, consisting of 75 patients (82 feet) after scarf osteotomy using headless compression screws, was used to compare clinical outcomes. **Results:** After fixation with allograft bone screws, the mean preoperative AOFAS score increased from 51.5 points preoperatively to 93.5 points one year after surgery. In radiological assessments, a continuous osseointegration with the remodeling of the bone screw was observed in all patients that finished follow-up. However, four metatarsal fractures occurred early postoperatively after fixation using allograft bone screws. There were only three material removal surgeries in patients treated with headless compression screws. **Conclusions**: Allograft bone screws display a safe fixation and are a biological alternative for scarf osteotomy. Enough distance between the screw and the proximal osteotomy should be ensured to avoid fractures.

## 1. Introduction

Various devices and materials are used for the fixation of osteotomies in forefoot surgery, as modern product processing offers an abundance of material forms and compositions [1,2]. This plurality offers intriguing possibilities, as new products might show different clinical profiles of the material advantages and drawbacks [3]. Standard metal implants come with excellent durability. They are, however, non-degradable, and material removal surgery might be indicated after implantation due to soft-tissue irritation and surgical site pain [4]. In contrast, biodegradable implants are gaining popularity in forefoot surgery. They display a continuous auto-degradation, which renders secondary removal unnecessary [5]. Polylactide and magnesium are commonly used as biodegradable implant materials. In these materials, reactions of the surrounding tissue with the implant material due to their degradation or corrosion, which might result in soft-tissue inflammation or osteolysis, are described [6,7]. Another well-established material is human bone allograft. Human cortical bone allograft shows a comparable stiffness to the original host bone and provides a steady profile of osseointegration without an expected lytic resorption [8]. In recent literature, a human allograft bone screw has been evaluated as osteotomy and arthrodesis fixation in multiple retrospective case series with satisfying clinical outcomes [9,10,11,12]. To further assess the advantages and disadvantages of an integrating material in forefoot surgery, the aim of this study was to prospectively observe the clinical safety and incorporation process of a novel human allograft bone screw (Shark Screw cut, Surgebright, Lichtenberg, Austria) in the treatment of hallux valgus using the scarf osteotomy and to compare clinical results to the usage of titanium headless compression screws (HCS).

## 2. Patients and Methods

**Figure 1:**
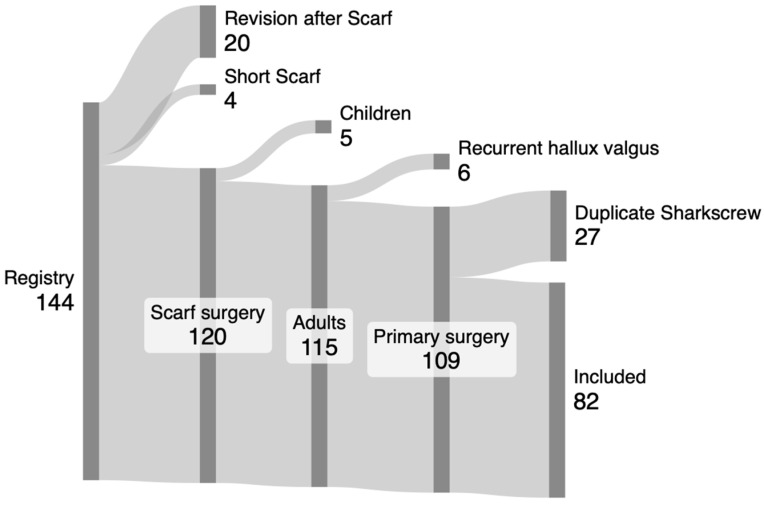
Patient inclusion chart for the retrospective control group. Eighty-two scarf surgeries for hallux valgus between 2017 and 2022 were included for retrospective assessment.

After approval of the local ethics committee (protocol code 1461/2018, date of approval: 18 June 2018), a prospective observational study was conducted at the Department of Orthopedics and Trauma Surgery, Medical University of Vienna. After application of inclusion criteria, which consisted of patients (1) with painful hallux valgus indicated to surgical correction with a scarf osteotomy (2) over 18 years, and potential exclusion criteria, which were (1) insufficient capacity of German language, (2) alcohol or drug abuse, (3) pregnant or breast-feeding women, (4) foreseeable compliance problems, (5) osteomyelitis, (6) skin lesions around the surgical site, and (7) revision surgery after hallux valgus surgery, twenty-one patients were enrolled in the study after giving written consent, and a scarf osteotomy was performed on thirty feet. Feet with additional forefoot pathologies were not excluded, and additional procedures were carried out on these patients. Eight feet received additional procedures together with a scarf osteotomy, twenty-two feet solely received a scarf osteotomy. Nineteen patients were female, and two patients were male. The mean patient age was 53 (30–70, SD 11) years (Table 1). The mean body mass index was 28 (20–37, SD 5.7). Additionally, a retrospective comparison group consisting of all patients (1) over 18 years that received (2) a scarf osteotomy using (3) HCS (4) between 2017 and 2022 at the (5) Department of Orthopedics and Trauma Surgery at the Medical University of Vienna was analyzed to compare the usage of allograft bone screws to HCS (Table 1). An in-house patient surgery report file search for all patients treated with scarf osteotomy for hallux valgus between 2017 and 2022 revealed 144 scarf surgeries, of which 75 patients with 82 feet were eligible for study inclusion (Figure 1). Of these 75 patients, 65 patients were female, and 10 patients were male.

### 2.1. Surgical Procedure and Postoperative Mobilization

**Figure 2:**
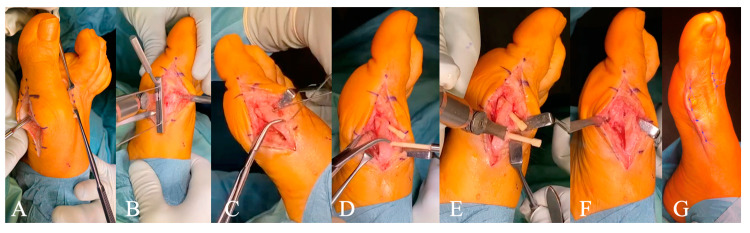
Surgical standard procedure (**A**) soft-tissue and capsule release (**B**) metatarsal-bone osteotomy (**C**) reposition with a clamp and K-wires (**D**) allograft screw implantation (**E**) screw-head removal on bone level (**F**) surgical result (**G**) wound closure.

All surgeries were standardized and performed at the same institution by the same attending surgeon (Figure 2). After skin incision medial of the first metatarsophalangeal joint and capsular incision, the bunion was removed. The lateral joint capsule was mobilized with a McGlamry elevator. A second incision was carried out between the first and second metatarsophalangeal joint, and a lateral capsular release and release of the contract tendon of the adductor hallucis muscle was performed. Afterwards, a Z-shaped osteotomy was performed using K-wires and jigs, and the distal first metatarsal bone was lateralized and compressed with a clamp and K-wires. Compression with a clamp was mandatory due to the design of the allograft bone screw as a simple set screw. Two threaded holes were drilled in the diaphyseal bone, and two 3.5 mm Shark Screw cut (Surgebright, Lichtenberg, Austria) were implanted. Primary stability of the allograft screw was subjectively evaluated by the implanting surgeon (SP) on a five-point scale (1 = excellent stability, 5 = very weak). The protruding screw heads were cut on bone level, and the metatarsal-bone step resulting from the bone reposition was removed. For closure, the overlaying joint capsule was removed, and the remaining capsule was tightly sutured. The skin was closed with Donati stitches. After observation in the recovery room, patients left the hospital and were referred to our outpatient clinic on the next day.

Postoperatively, patients were usually mobilized with shoes giving forefoot support for six weeks. Hallux valgus socks were usually put on after wound healing two weeks after surgery until six weeks after surgery. A walker orthosis was put on for two weeks after surgery to ensure safe bone healing in patients with poor bone quality or slim metatarsal bones. In cases of metatarsal fractures and very poor bone quality, patients received a white plaster cast for six weeks after the event.

### 2.2. Follow-Up Visits and Outcome Scores

**Figure 3:**
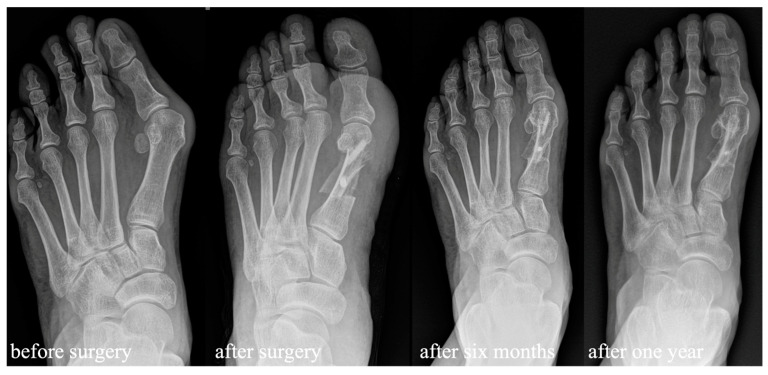
Radiologic pre- and postoperative dorsoplantar imaging with partial remodeling six months after surgery and full remodeling one year after surgery.

Standardized study visits using case report forms were carried out before surgery and two weeks, six weeks, six months, and one year after surgery. The follow-up visits consisted of a patient assessment including the original American Orthopedic Foot and Ankle Society forefoot rating system (AOFAS) and a visual analogue scale for pain on all follow-up visits as well as a satisfaction scale with the surgery itself at the follow-up examinations 6 months and 1 year after surgery [13]. Visual analogue scales were measured on a 10-point scale with 0 indicating no pain at all and 10 indicating maximum pain. Satisfaction with surgery was measured on a 10-point scale with 0 indicating full satisfaction and 10 indicating no satisfaction with surgery at all. Range of motion of the first metatarsophalangeal joint was assessed with a goniometer on all follow-up visits and further subclassified for statistical analysis according to the metatarsophalangeal range of motion of the AOFAS score, with 1 = normal range of motion (75° or more), 2 = medium restricted (30°–75°), and 3 = severely restricted (<30°). Patients were asked about any adverse events, such as fever, sudden pain events, or falls, since the last outpatient clinic visit. On all follow-up visits, a surgical site inspection and a sensibility check were performed. Weight-bearing dorsoplantar and lateral X-rays were obtained six weeks, six months, and one year after surgery (Figure 3). X-rays two weeks after surgery were performed without weight bearing. 

The standard follow-up visits for the retrospective control group consisted of a visit at the outpatient clinic one day after surgery and thereafter weekly for 6 weeks. Further visits at the outpatient department were recommended only if necessary. X-rays were taken before and immediately after surgery as well as 6 weeks after surgery. All electronic patient charts were screened for further revision surgeries on the same foot performed in other national public hospitals using the Austrian electronic health record software (ELGA, version 2.8).

### 2.3. Measurement and Outcome Scores

#### Radiological Measurements

**Figure 4:**
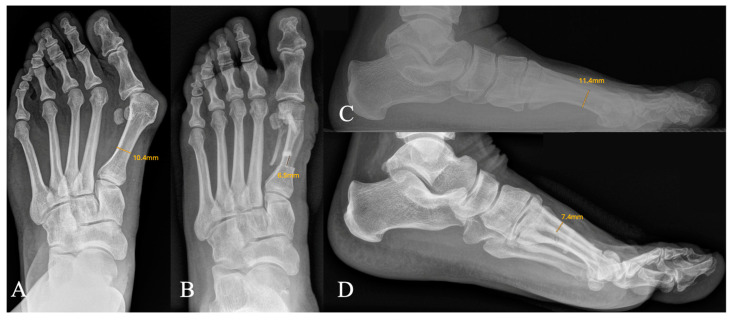
Fracture analysis of patients treated with allograft bone screws in scarf surgery. (**A**) Preoperative dorsoplantar X-ray showing the minimal diaphyseal width (**B**) postoperative dorsoplantar X-ray showing distance from the proximal screw to the proximal osteotomy (**C**) preoperative lateral X-ray showing the minimal diaphyseal metatarsal depth (**D**) postoperative lateral X-ray showing the distance between the proximal plantar osteotomy and the dorsal diaphyseal metatarsal cortical bone.

X-ray measurement of the hallux valgus angle (HVA) and intermetatarsal angle (IMTA) was performed on weight-bearing X-rays. The HVA was defined as the angle between the first metatarsal bone and the first proximal phalanx; whereas, the IMTA was defined as the angle between the first and second metatarsal bone. Osseointegration of the allograft bone screw was defined as bony ingrowth of the allograft bone screw in X-rays without newly developed osteolyses or radiolucencies around the bone-to-implant interface. Screw remodeling was defined as vanishing of the allograft bone screw, with full remodeling, indicating that the screw body was not delimitable from the surrounding host bone in standard X-rays. A residual sclerosis at the place of the screw was also considered as full remodeling. Fracture analyses included measurements of the minimal diaphyseal metatarsal width and depth before surgery as well as the depth of the proximal bone bridge at the level of the proximal osteotomy in lateral X-rays and the distance between the allograft bone screw and the proximal osteotomy in dorsoplantar X-rays immediately after surgery (Figure 4).

### 2.4. Statistical Analysis

A Sankey diagram was used for depicting patient inclusion for the retrospective control group (https://sankeymatic.com/build/, accessed on 15 June 2024). Descriptive statistics were performed to depict means, frequencies, and ranges. Longitudinal changes of metric parameters of patients during the follow-up period, such as differences in radiographic X-ray measures, AOFAS, or VAS scores, were analyzed using paired sample *t*-tests. Mann–Whitney U-tests were performed for nonparametric analyses of postoperative fractures after bone-screw implantation. Fisher’s exact test was performed to analyze postoperative fractures and wound healing disorders between groups. A *p* value < 0.05 was considered significant. 

## 3. Results

### 3.1. Clinical and Radiological Outcomes after Scarf Osteotomy Using Allograft Bone Screws

Of the 21 patients and 30 feet enrolled using allograft bone screws, 16 patients and 25 feet completed the follow-up until one year after surgery. (Table 1).

All five patients lost to the follow-up were successfully contacted after drop-out. Three of them were very satisfied with the surgery but were cautious to come to the hospital due to the COVID-19 pandemic and finished follow-up six weeks after surgery. Two further patients received a reoperation due to a recurrent hallux valgus or hallux varus in the follow-up period and, thus, did not finish the follow-up. The mean AOFAS steadily increased from before surgery (51.5 SD 11; range 24–69) to one year after surgery (93.5 SD 6.3; range 78–100, *p* = 0.001, Table 2). 

Patients were very satisfied in the postoperative course six months (9.4 SD 1.5; 2–10) and one year (9.5 SD 0.9; range 6–10) after surgery (*p* = 0.5). 

**Figure 5:**
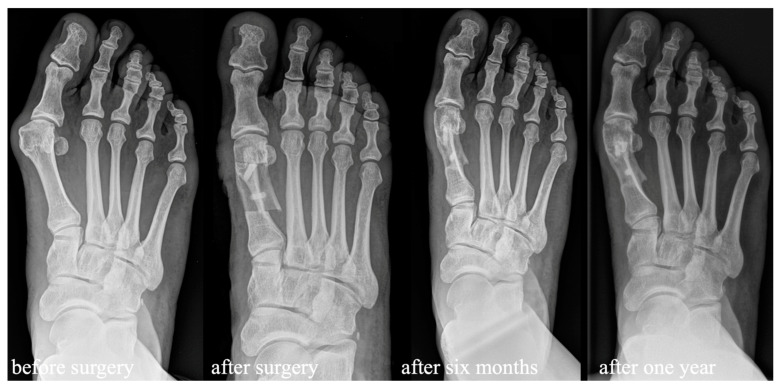
Radiologic pre- and postoperative imaging displaying a continuous screw integration with a full remodeling and residual sclerosis one year after surgery.

In X-rays, osseointegration without osteolyses or radiolucencies around the bone screw was observed in all patients at the follow-up visit 6 weeks after surgery. Full remodeling of the allograft bone screw was observed in 9 feet after six months and in all the other 17 feet available after one year (Figure 5).

**Figure 6:**
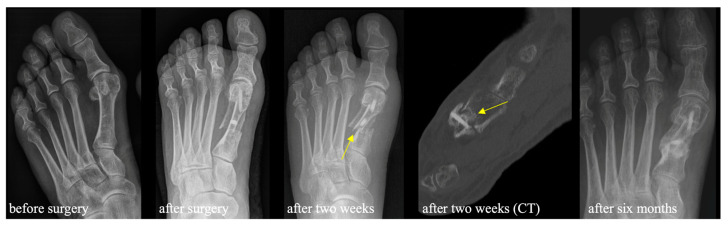
Radiologic imaging of a patient showing a fracture at the level of the proximal osteotomy after a fall two weeks after surgery (arrows). The patient received a white plaster cast for six weeks following the fracture. At six months after index surgery, the fracture was healed, while the allograft-screw-thread blur without osteolysis was a sign of osseointegration and partial remodeling.

Intraoperative subjective primary screw stability was satisfactory, with a score of 1.4 (SD 0.6; range 1–3) on a five-point scale (1 = excellent, 5 = very weak). Regarding complications after surgery, four patients suffered from superficial wound healing disorders, which were resolved with simple oral antibiotic treatment. Paresthesia at the lateral side of the first toe and the medial side of the second toe was reported in twelve patients six weeks after surgery, but only three patients had a persistence of this paresthesia until one year after surgery. Five patients suffered from a fall in the early postoperative period, whereof four patients presented with a fracture at the proximal first metatarsal bone at our outpatient clinic with a mean of 13.5 (12–15) days after surgery (Figure 6). 

All four patients received a white plaster cast (n = 3) or walker orthosis (n = 1) for six weeks thereafter. A sub-analysis comparing patients with postoperative metatarsal fractures against patients without fracture after bone-screw implantation showed that patients with fractures had a lower distance from the proximal screw body to the proximal cut of the scarf osteotomy in postoperative dorsoplantar X-rays, and their longitudinal bone cuts were placed more dorsally in lateral X-rays (Table 3). 

Regarding revision surgeries, one of the patients after fracture suffered from a recurrent hallux valgus and received a Lapidus arthrodesis ten months after index surgery. Another patient developed a progressive collapsing foot deformity and received a hallux varus correction osteotomy six months after index surgery. There were no intraoperative or postoperative material-specific complications, such as screw failure, screw loosening, loss of fixation, or osteolysis around the screw.

### 3.2. Comparison of the Usage of Allograft Bone Screws to HCS for Scarf Fixation

**Figure 7:**
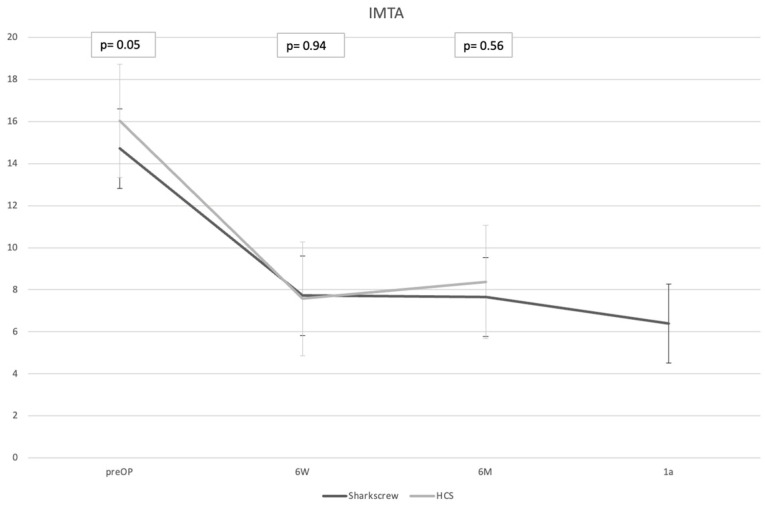
Comparison of IMTA angles (y-axis, degrees) between both screw types over time (x-axis; 6W = 6 weeks, 6M = 6 months, 1a = 1 year).

**Figure 8:**
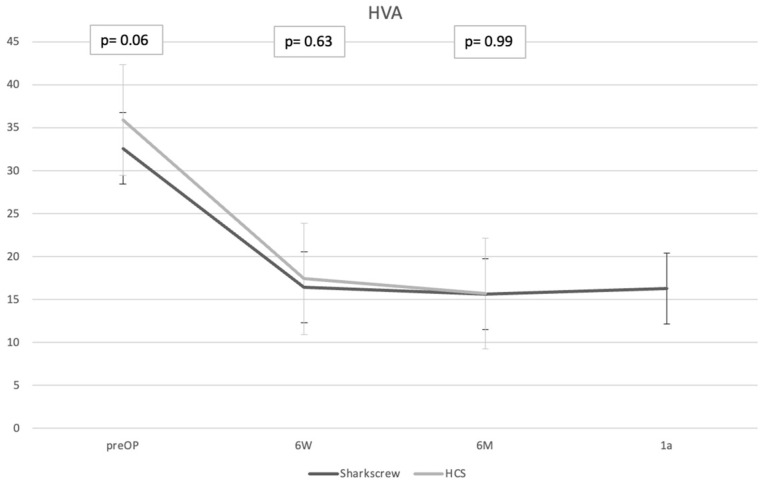
Comparison of HV angles (y-axis, degrees) between both screw types over time (x-axis; 6W = 6 weeks, 6M = 6 months, 1a = 1 year).

Patients treated with allograft bone screws had a longer mean postoperative follow-up and a more restrictive postoperative immobilization in comparison to patients treated with HCS. The occurrences of complications and revision surgeries were similar between groups (Table 1). Screw removal surgeries were only indicated in three patients after HCS implantation with a mean of 16 (4–38) months after initial surgery. In X-rays, the postoperative deformity correction assessed by IMTA (Figure 7) and HVA (Figure 8) were comparable between groups.

## 4. Discussion

Modern implants expand the armamentarium of surgeons to further improve patient outcomes and to address complex indications. Allograft bone screws show an intriguing set of material-specific characteristics due to their biocompatibility, osseointegrative behavior, and remodeling. However, there is a lack of prospective data observing surgical outcomes and the incorporation process of this novel implant. We found a high level of screw to host bone integration in a one-year prospective observational study. On the other hand, caution should be taken in cases of slender metatarsal bones in connection with slim proximal bone bridges after scarf osteotomy to avoid metatarsal fractures. This information should help treating surgeons in indicating and handling allograft bone screws in scarf osteotomies, as well as to further assess the screw’s profile.

### 4.1. Clinical and Radiological Outcomes after Scarf Osteotomy

Patients reported satisfactory clinical outcomes one year after scarf osteotomy using allograft bone screws as fixation. This finding was according to the literature, as the literature shows comparable clinical outcomes after both scarf osteotomy and after the use of other biodegradable implant materials in forefoot surgery [14,15,16]. However, metatarsal fractures occurred in 4 of 30 feet in the early postoperative period. This is a higher percentage compared to the literature, as Clee et al. described metatarsal fractures in 4.6% of patients in a larger multicenter review [17]. A higher incidence of metatarsal fractures might be related to intraoperative adjustments to ensure fixation of the bone set screw, with a tendency to a more dorsal osteotomy to ensure enough bone volume for screw fixation in the plantar bone. In retrospect, these adjustments possibly would not have been necessary, due to the satisfactory intraoperative primary stability of the bone screw. A high primary stability might be attributed to the high number of thread turns of the implant, which allows for a high surface area and bone-to-bone contact and might additionally facilitate osseointegration and stability. Even after metatarsal fracture, the allograft bone screw maintained the fixation of the osteotomy, indicating a sufficient structural durability of the screw, even in unstable bony conditions of the forefoot. Additionally, the allograft bone screw has a 3.5 mm width body, which is comparable to other metal implants used in forefoot surgery, and the screw head is not protruding and cut off after implantation above bone level. Although patients suffering from a fracture in this study reported not wearing their forefoot supplying shoe at the time of their fall, it is unknown whether the fractures are because of the event or result, and at least an increased susceptibility should be assumed. A sufficient dorsal-bone bridge and enough distance between the screw and the proximal osteotomy should be intraoperatively ensured to lower the fracture risk. We further recommend the use of more restricting orthoses, such as walker boots or white plaster casts, especially in patients with weak bone quality or slim metatarsal bones. Additionally, oblique X-rays should be made in early follow-up visits to detect non- or minimally displaced metatarsal fractures. In selected cases, the use of slimmer conventional metal screws might be favorable to potentially lower the fracture risk; although, more studies are needed to evaluate this topic. 

Screw remodeling was observed in all patients during the first postoperative year. These results were according to the literature, where a full osseointegration and remodeling of the screw is continuously reported [11,18]. There were no material-specific adverse events observed clinically and in X-ray follow-up in this study. In comparison, Delsmann et al. reported osteolysis and radiolucent zones in X-rays after the use of magnesium-based biodegradable compression screws for fixation of osteotomies, fractures, and osteochondral defects in 27 of 29 patients at the first follow-up; albeit, the radiolucent zones were self-limiting and disappeared without complications in further follow-up examinations [6]. Haslhofer et al. reported 55.6% of patients with osteolysis after using the same magnesium-based biodegradable compression screw, with 22% of patients suffering from material failure for treatment at a level-1 trauma center [19]. By contrast, adverse material reactions have not been reported in multiple case series after the use of allograft bone screws [18]. As the host bone accepts and integrates the allograft cortical bone screw without displaying signs of surrounding tissue reactions, this unique material profile might help to further reduce adverse reactions in the usage of bioabsorbable materials. 

The field of surgical indications for this new implant type warrants further exploration. Allograft bone screws may be especially promising as salvage implant in patients with non-union in foot and ankle or hand surgery. Other indications that might benefit from this implant type include fractures of bones and cartilage where perfusion is limited and osteoconductive potential is needed, such as in the fixation of scaphoid fractures or osteochondritis dissecans [9]. Further studies are needed to evaluate this implant in these indications.

### 4.2. Evaluation of Benefits and Disadvantages in Comparison to HCS in Scarf Surgery

In comparison to the use of allograft bone screws, HCS showed similar results regarding deformity correction in X-rays, with a low number of complications and revision surgeries in the postoperative follow-up. Prior analyses of material removal surgeries after distal metatarsal osteotomies for hallux valgus correction showed a high discrepancy of 2.9% to 27.8% [4,20]. Only 4% of patients treated with HCS required material removal in combination with cheilectomy or soft-tissue arthrolysis due to first metatarsophalangeal stiffness in this study. It seems reasonable that a low incidence of soft-tissue irritation using HCS should be attributed to the subsidence of the screw head into the metatarsal bone. As one propagated advantage of the use of allograft bone screws is the redundancy of material removal surgeries due to the screw remodeling over time, it is debatable whether an inherent redundancy is relevant in metatarsal osteotomies based on the already excellent results of HCS in this study.

### 4.3. Limitations

This study has several limitations. First, the postoperative follow-up was limited to one year, and there is no available outcome data after this period. A retrospective case series of Bock et al. shows 30% of patients suffering from recurrent hallux valgus after scarf osteotomy ten years after surgery. It should be assumed that patients with recurrent hallux valgus are not displayed in this study to the full extent. However, even in revision surgeries, patients benefit from the allograft bone screw due to its integrating character and the redundancy to explant the screws during surgery. Although follow-up computer tomography or magnetic resonance imaging would have given additional information, especially regarding time points of early osseointegration, the screw showed clear osseointegration and remodeling in X-rays in all patients of this study, and thus, advanced imaging methods were not used to determine osseointegration in this surgical indication. 

The retrospective outpatient clinic data of patients receiving headless compression screws was generally not detailed enough to retrospectively assess functional outcomes and AOFAS scores. However, the main reason to implement the retrospective HCS group was primarily not to analyze functional outcomes, but to assess incidences of surgical complications and revisions. 

## 5. Conclusions

Allograft bone screws display a safe fixation and are a biological alternative for scarf osteotomy. The bone screw’s constant osseointegration and remodeling are primary benefits compared to conventional HCS; although, material removal rates of HCS were already low. In cases of slim intraoperative dorsal-bone bridges, enough distance between the screw and the proximal osteotomy should be ensured to avoid fractures.

## Figures and Tables

**Table 1:** Comparison of surgery-specific parameters of patients undergoing scarf osteotomy using allograft bone screws versus headless compression screws. Age at surgery and follow-up time shown as mean (median, range).

Parameter	Allograft Bone Screw (n = 30)	Headless Compression Screw (n = 82)	*p*
Age at surgery	53 (52, 30–70) years	57 (59, 22–79) years	0.075
Follow-up time	10.7 (12, 1.5–13) months	6.9 (1.5 months, 1 day–6.3 years) months	<0.001
Immobilization			
Biocomfort shoe alone	19 (63%)	79 (96%)	<0.001
Plaster cast	5 (17%)	3 (4%)	0.018
Walker orthosis	6 (20%)	0 (0%)	<0.001
Complications after surgery			
Fracture	4 (13%)	4 (5%)	0.21
Wound healing disorder	4 (13%)	6 (7%)	0.45
First ray revision surgery	2 (7%)	6 (7%)	0.91
Recurrent hallux valgus	1 (3%)	2 (2%)	0.8
Hallux varus	1 (3%)	1 (1%)	0.45
Material removal	0 (0%)	3 (4%)	0.29

**Table 2:** Clinical results in the first postoperative year after scarf osteotomy using allograft bone screws. Shown *p*-values represent the paired *t*-test results between the parameters before surgery and one year after surgery, except for the satisfaction with surgery, where the *p*-values represent the paired *t*-test results six months and one year after surgery.

Parameter (n = 30)	Pre-Surgery	Two Weeks	Six Weeks	Six Months	One Year	*p*
AOFAS score	51.5 (11; 24–69)	59 (10; 35–75)	70 (8; 50–87)	89 (13; 39–100)	93.5 (6; 78–100)	0.001
Visual analogue scale (pain)	5 (2; 1.5–9.5)	3 (2; 0–8)	1 (1; 0–5)	1.5 (2; 0–7.5)	0.5 (1; 0–3)	0.001
Satisfaction with surgery	/	/	/	1 (1.5; 0–8)	0.5 (1; 0–4)	0.5
Range of motion	1.5 (0.5; 1–2)	2.5 (1; 1–3)	2 (1; 1–3)	1.5 (1; 1–3)	1 (0.5; 1–2)	0.5
Dorsal extension	38 (7; 30–45)	18 (9; 10–30)	24 (13; 5–50)	33 (10; 10–55)	39 (12; 20–60)	0.4
Plantar flexion	40 (11; 20–65)	19 (8; 10–30)	23 (11; 5–40)	32 (14; 5–55)	36 (11; 10–50)	0.1

**Table 3:** Fracture analysis of patients treated with allograft bone screws.

Parameter	Fracture (n = 4)	No Fracture (n = 26)	*p*
Age at surgery			
Minimal diaphyseal width	13.3 (±0.96) mm	13.3 (±0.93) mm	0.98
Minimal diaphyseal depth	13.3 (±1.2) mm	13.7 (±0.98) mm	0.78
Distance proximal osteotomy to bone screw	4.3 (±1.5) mm	5.8 (±1.6) mm	0.044
Depth of dorsal-bone bridge	5.0 (±0.82) mm	7.4 (±0.90) mm	<0.001

## Data Availability

Study data are available from the corresponding author on reasonable request.
